# Vitellogenin genes are transcribed in *Culex quinquefasciatus* ovary

**DOI:** 10.1590/0074-02760220143

**Published:** 2023-07-17

**Authors:** Alexandre S Moura, André Luis Costa-da-Silva, Pedro S Peixoto, Ceres Maciel, André F Cardoso

**Affiliations:** 1 Universidade de São Paulo, Instituto de Ciências Biomédicas, Departamento de Parasitologia, São Paulo, SP, Brasil; 2 Florida International University, Sciences Institute, Department of Biological Sciences & Biomolecular, Miami, FL, USA; 3 Universidade de São Paulo, Instituto de Matemática e Estatística, Departamento de Matemática Aplicada, São Paulo, SP, Brasil; 4 Universidade do Estado de Mato Grosso, Departamento de Ciências Biológicas, Tangará da Serra, MT, Brasil

**Keywords:** Culex quinquefasciatus, oogenesis, vitellogenin transcription profile, *in situ* hybridisation

## Abstract

**BACKGROUND:**

*Culex quinquefasciatus*, a cosmopolitan, domestic, and highly anthropophilic mosquito, is a vector of pathogenic arboviruses such as West Nile virus and Rift Valley virus, as well as lymphatic filariasis. The current knowledge on its reproductive physiology regarding vitellogenin expression in different tissues is still limited.

**OBJECTIVES:**

In this study, we analysed the transcriptional profiles of vitellogenin genes in the fat body and ovaries of *C. quinquefasciatus* females during the first gonotrophic cycle.

**METHODS:**

*C*. *quinquefasciatus* ovaries and/or fat bodies were dissected in different times during the first gonotrophic cycle and total RNA was extracted and used for reverse transcription polymerase chain reaction, quantitative real time*-*PCR, and *in situ* hybridisation.

**FINDINGS:**

We confirmed the classical descriptions of the vitellogenic process in mosquitoes by verifying that vitellogenin genes are transcribed in the fat bodies of *C. quinquefasciatus* females. Using RNA *in situ* hybridisation approach, we showed that vitellogenin genes are also transcribed in developing ovaries, specifically by the follicle cells.

**MAIN CONCLUSIONS:**

This is the first time that vitellogenin transcripts are observed in mosquito ovaries. Studies to determine if Vg transcripts are translated into proteins and their contribution to the reproductive success of the mosquito need to be further investigated.

*Culex (C.) quinquefasciatus* is an important vector of *Wuchereria bancrofti*, the aetiologic agent of the lymphatic filariasis, and can transmit a variety of encephalitis-causing viruses, including West Nile Virus.^1^ ^,^ ^2^ ^,^ ^3^ It is also responsible for great nocturnal nuisance^4^ ^,^ ^5^and severe reactions in patients allergic to mosquito bites.^6^ ^,^ ^7^ These aspects demonstrate the importance of controlling *C. quinquefasciatus* populations and the transmission of their associated pathogens.

Like all oviparous animals in which the embryonic development occurs outside the maternal body, the mosquito’s eggs contain all the nutrients required for the growing embryo.

According to the classical descriptions of the vitellogenic process in mosquitoes, yolk protein precursors are synthesised by fat body trophocytes, secreted to the haemolymph, and incorporated by developing oocytes through receptor-mediated endocytosis^8^ ^,^ ^9^ ^,^ ^10^ ^,^ ^11^ ^,^ ^12^ ^,^ ^13^ Intake of yolk precursors is facilitated by the transient opening of intercellular channels in the follicular epithelium^14^ ^,^ ^15^ that envelopes the ovarian follicle. Ovarian follicles comprise one oocyte and a set of nurse cells responsible for the synthesis of ribosomes and RNA that are transferred to the oocyte through cytoplasmic bridges.^8^ ^,^ ^14^ Follicle cells synthesise the set of eggshell proteins.^15^ ^,^ ^16^

However, Anderson and Telfer, already in 1969, working with the saturniid moth *Hyalophora cecropia*, demonstrated that in some insects, biosynthesis of yolk proteins also occurs in the ovarian follicular cells.^17^ In 1980, Postlethwait and colleagues showed the biosynthesis of vitellogenin by the ovaries of *Drosophila melanogaster* ^18^ and in 1982 Brennan and colleagues reported that around 30% of the vitellogenins of this insect is synthesised in the ovaries during the later stages of oogenesis.^19^ Similar results were reported in 1985 by de Bianchi and co-workers in *Musca domestica* and these authors suggested that there is a metabolic balance between the synthesis of these proteins by the follicular epithelium and the resorption of nurse cells observed at this stage.^20^

Despite these and other descriptions of vitellogenin biosynthesis in the ovaries of insects [Coleoptera,^21^ Lepidoptera^22^ and Hemiptera^23^], to our knowledge there are no reports of vitellogenin biosynthesis in the ovaries of mosquitoes (Diptera, Culicidae).

Although the production of vitellogenin by *C. quinquefasciatus* fat body was already characterised,^14^ the involvement of mosquito ovaries in the transcription of vitellogenin genes has never been described. In this study we present the transcriptional profiles of *C. quinquefasciatus* vitellogenin genes in the fat body and ovaries of females over the first gonotrophic cycle. Our results confirm abundant transcription of Vitellogenin (Vg) genes in the fat bodies and also show the accumulation of Vg transcripts in the follicular epithelium of *C. quinquefasciatus* ovaries.

## MATERIALS AND METHODS

*Ethics* - All procedures were approved by the Ethics Committee on Animal Experiments of Institute of Biomedical Sciences from University of São Paulo (CEUA n^o^ 103).

*Animals* - *Culex quinquefasciatus* [PIN strain - ^24^] were raised under a photoperiod of 12 h dark-12 h light at 27ºC, 70-80% relative humidity. Larvae were fed with ground fish food (Sera^®^vipan, Germany), and adults were fed on 10% sucrose solution *ad libitum*. When necessary, 4-5 days old adult females were fed on Balb/c mice anaesthetised with 0.3 mg/kg of xylazine hydrochloride (Calmiun, Agner União, Brazil) plus 30 μg/kg of acepromazine (Acepran, Univet SA, Brazil).

*RNA extraction* - Pools of ten female mosquitoes in different timepoints were used for RNA extraction. Three pools were used for the experiments described below. Ovaries and fat bodies (*i.e.*, abdomens free of gut), Malpighian tubules and ovaries^25^ were dissected from adult females fed on sucrose (SUC) and at 12, 24, 36, 48, 60, 72, and 84 hours post blood meal (PBM). Total RNA was extracted using Trizol^®^ (Invitrogen, USA) according to the manufacturer’s instructions. Residual genomic DNA was removed from RNA samples by incubation with DNase I, Amp Grade (Invitrogen, USA). Total RNA was quantified on a NanoDrop 2000 UV-Vis spectrophotometer (Thermo Scientific, USA).

*Oligonucleotide design* - Oligonucleotide primers for reverse transcription polymerase chain reaction (RT-PCR) amplification, detection and quantification were designed using the Primer3 program (http://frodo.wi.mit.edu/.^26^ All oligonucleotides were designed using the transcript sequences available in VectorBase as template (Table) and synthesised by Exxtend (São Paulo, Brazil) (Table).

**TABLE:** Gene-specific oligonucleotides used for polymerase chain reaction (PCR) and real-time PCR (RT-PCR) transcript analyses

Gene ID	VectorBase accession numbers	Primer ID	Oligonucleotide sequence
Vitellogenin 1	CPIJ010190	Vg1-F	5’CCGGTACTACAACCACAACG3’
	CPIJ010191	Vg1-R	5’TCTTCTCTTCCTCGGAGCTG3’
Vitellogenin 2	CPIJ001357	Vg2-F	5’CCGGTACTACAACCACAACG3’
	CPIJ001358	Vg2-R	5’GTCGCTGTGGTACACGATGA3’
Actin	CPIJ016462	Act-F	5’ACAGGTCATCACCATCGGTA3’
		Act-R	5’TCCTTCTGCATACGATCAGC3’
Ribossomal protein 49 gene (Rp49)	CPIJ001200	Rp49-F	5’AGGTATCGACAACCGAGTGC3’
		Rp49-R	5’ACAATCAGCTTGCGCCTTCTT3’

*RT-PCR* - RT was carried out with 2 μg of total RNA primed with 500 ng of oligo dT (Invitrogen, USA) and the SuperScript^®^ II first-strand synthesis system (Invitrogen, USA) as described by the manufacturer.

PCR was performed with 1 μL of complementary DNA (cDNA) as template and 0.4 μM of each primer (Table). Reactions were performed in a T3 Biometra^®^ (Biometra, Germany) thermocycler as follows: 2 min at 94ºC, followed by 40 cycles at 94ºC for 20 s, 55ºC for 20 s, and 72ºC for 40 s. Amplified products were resolved on 1.0% agarose gels, stained with Gel Red^®^ (Uniscience, Brazil), and visualised on an Image Quant 300 (GE Life Sciences, USA).

*Quantitative real time-PCR* - Quantitative real time*-*PCR (qRT-PCR) was performed using a StepOne™ Real-Time PCR System (Applied Biosystems, USA) in 96-well optical reaction plates (Applied Biosystems, USA). 1 μL (approximately 100 ng) of cDNA template, 0.6 μM of each primer (Table), and 8 μL of Maxima SYBR^®^ Green mix (Thermo Scientific, USA) were mixed for each 16 μL reaction. The thermal cycling program was: 10 min at 95ºC, followed by 40 cycles at 95ºC for 15 s, 57ºC for 1 min, and 60ºC for 1 min. The threshold cycle (Ct) was normalised according to the transcription levels of rp49 ribosomal protein (rp49, CPIJ001220), a constitutively expressed gene used as reference.^27^ The ribosomal protein rp49 gene (rp49) had an average Ct of 19.01 and 19.78 in the ovary and fat body samples, respectively. The relative expression of genes of interest was calculated using the 2^-∆∆C^T method.^28^ The expression levels in adult females fed on SUC was used as the reference condition. Data correspond to three independent biological samples. Each biological sample was analysed in technical triplicate.

*Statistical analysis* - The gene expression profiles were tested for normality distribution, revealing the need of nonparametric statistical analysis. Boxplots were used to represent the expression data and Kruskal-Wallis tests were used to evaluate the expression variation through timepoints. Dunn’s multiple comparison post hoc tests were used to compare the expression at a given time with the standard condition (SUC). All tests considered a significance level of 0.05.

*In situ hybridisation* - Ovaries 36 h PBM were dissected in 1x PBS, pH 7.4. Fixation and *in situ* hybridisation were performed as described by Juhn and James.^29^ Gene transcripts of interest were hybridised using DNA probes labelled with biotin, and detection was performed using anti streptavidin-biotin antibody conjugated with alkaline phosphatase enzyme (Life Technologies, USA). Staining was done using NBT/BCIP (KPL, USA) to detect the alkaline phosphatase activity. The biotin-labelled probes were produced by PCR using Taq Platinum polymerase (Life Technologies, USA). The PCR conditions are the same described above (RT-PCR).

Single ovarioles were mounted on a slide glass and were visualised in a DMLB microscope (Leica, Germany) coupled to a DFC 320 camera (Leica, Germany).

## RESULTS AND DISCUSSION

Insect vitellogenins may be classified into four classes: (1) vitellogenins that are synthesized in the fat body in a sex-specific manner and uptaken by the oocytes; (2) vitellogenins that are produced both in the female fat body cells and ovarian follicle cells in a sex-specific manner; (3) vitellogenins that are synthesized in the ovarian follicle cells and uptaken into the oocytes in a sex-specific manner and (4) vitellogenins that are synthesized in the fat body in a sex non-specific manner and secreted into the hemolymph.^30^

*Vitellogenin transcripts accumulate in C. quinquefasciatus ovaries* - Four sequences of the *C. quinquefasciatus* vitellogenin genes, all of them annotated as vitellogenin A1-precursor are deposited in VectorBase (https://www.vectorbase.org), the database of invertebrate vectors of human pathogens. The four genes were designated CpVg1a, CpVg1b, CpVg2a and CpVg2b by Chen and colleagues.^31^ The two CpVg families CpVg1 and CpVg2 share ~ 65% nucleotide identity. The two genes in subfamily CpVg1a and CpVg1b share high nucleotide identity (98.9%), while CpVg2a and CpVg2b are 98.4% identical. Although this manuscript refers to the same genes described by Chen et al.,^31^ we will use CqVg instead of CpVg, to refer to *C. quinquefasciatus* vitellogenin genes. Four full length Vg genes also were identified in *Culex tarsalis*, a closely related species. The *C. tarsalis* Vg genes were designated CtVg1a, CtVg1b, CtVg2a and CtVg2b. Sequence analysis indicated that CtVg1 and CtVg2 families shared 64.3%-65.5% nucleotide identity. CtVg1a and CtVg1b shared extremely high nucleotide identity (98.1%), while CtVg2a and CtVg2b shared 97.0% identity.^31^

**Fig. 1:**
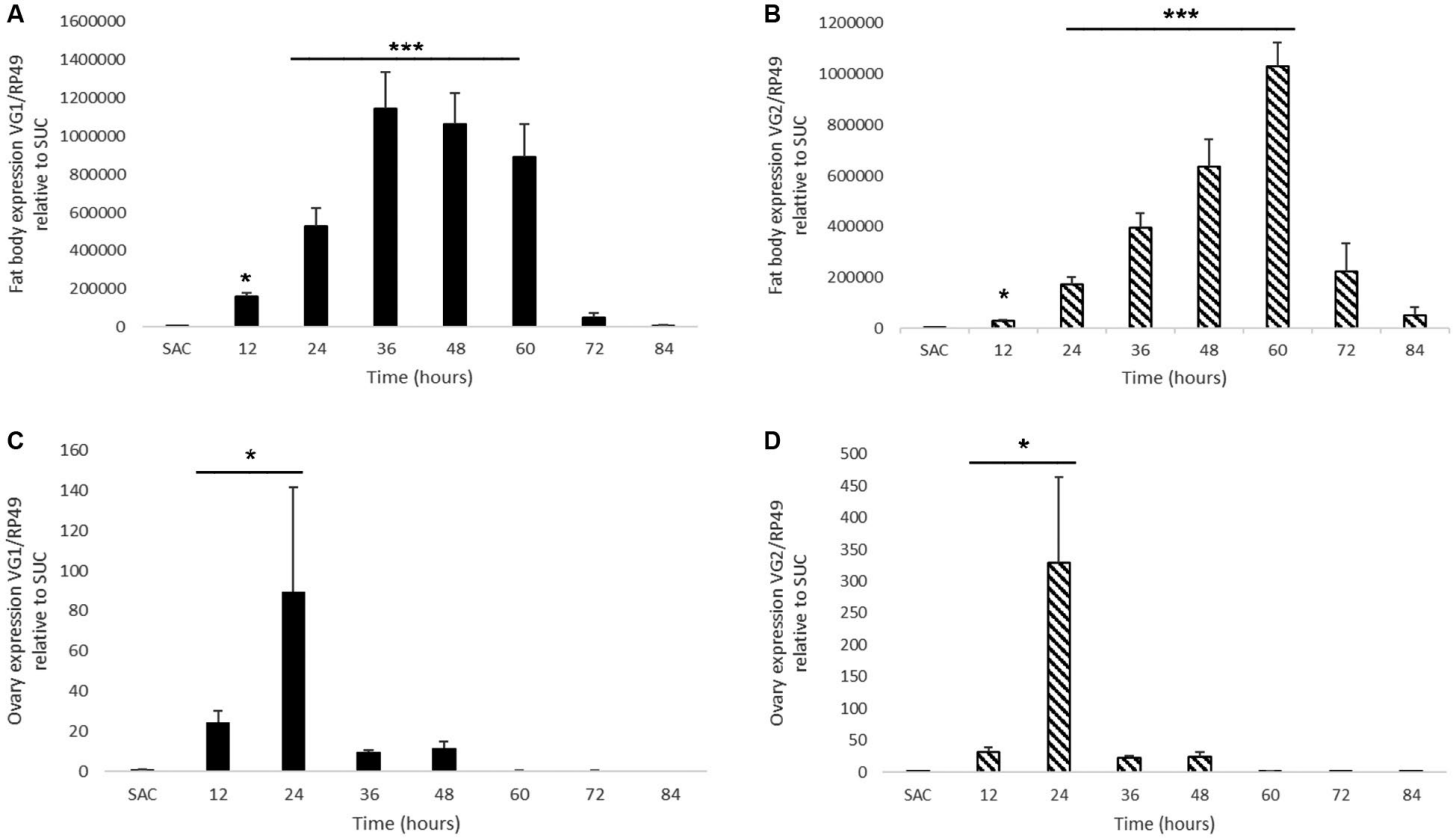
expression profile of *Culex quinquefasciatus* vitellogenin 1 gene (CqVg1) and vitellogenin 2 gene (CqVg2) transcripts during the first gonotrophic cycle (12 h, 24 h, 36 h, 48 h, 60 h, 72 h, and 84 h are times post blood meal). A: CqVg1 expression in fat body; B: CqVg2 expression in fat body. C: CqVg1 expression in ovaries; D: CqVg2 expression in ovaries. The bars represent the means of three independent experiments [means ± standard error of the mean (SEM)]. Asterisks denote quantitative real time-polymerase chain reaction (qRT-PCR) data with a statistically significant difference when compared to sugar-fed females (SUC). Kruskall-Wallis Test with Dunn’s multiple comparison vs. SUC, *p < 0.05; ***p < 0.001.

**Fig. 2:**
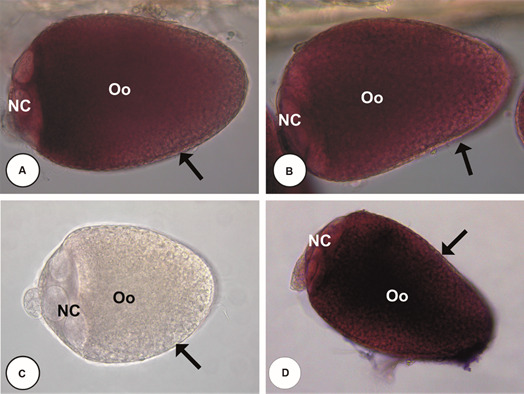
*in situ* hybridisation of vitellogenin mRNAs in *Culex quinquefasciatus* ovarian follicle 36 h post blood meal (PBM). (A): *C. quinquefasciatus* vitellogenin 1 gene (CqVg1) transcript (CPIJ010190/CPIJ010191); (B): *C. quinquefasciatus* vitellogenin 2 gene (CqVg2) transcript (CPIJ001357/CPIJ001358); (C): negative control (without substrate); (D): ribosomal protein 49 gene (Rp49 - positive control). NC: nurse cells; Oo: oocyte; arrow: follicular epithelium. X 400.

Because of the high identity between the Vg sequences [Supplementary data (Fig. 1)], it was not possible to design specific primers for each of the four Vg genes deposited in the VectorBase. Consequently, three primers were designed: a forward one which is conserved between the selected regions of all four CqVg genes and two different reverse primers which co-amplify either CPIJ010190 and CPIJ010191 (CqVg1) or CPIJ001357 and CPIJ001358 (CqVg2), respectively [Supplementary data (Fig. 2)].

Our data show that vitellogenin genes are abundantly transcribed in *C. quinquefasciatus* fat bodies following a blood meal, similar to the results reported for other mosquito species (*Aedes aegypti*,^32^ ^,^ ^33^ *Anopheles gambiae* ^34^ and *Anopheles aquasalis* ^35^) [Supplementary data (Fig. 3)].

However, different from other mosquitoes, our results also evidenced transcription of vitellogenin genes in the ovaries of *C. quinquefasciatus* following a blood meal [Supplementary data (Fig. 3)].

To further investigate the dynamics of Vg transcripts accumulation in *C. quinquefasciatus* during the first gonotrophic cycle, qRT-PCR was performed. Fat bodies had no detectable Vg transcripts prior to females taking a blood meal, and showed abundant CqVg1 and CqVg2 transcripts from 12 h until 60 h PBM (Fig. 1A-B). Vg transcription declined afterwards to very low or undetectable levels, consistent with the end of the gonotrophic cycle.

The qRT-PCR data also show that Vg transcripts are present in the ovaries of *C. quinquefasciatus* following a blood meal, albeit at a lower level than those measured in the fat bodies. CqVg1 and CqVg2 transcripts are detected in the ovaries between 12 h and 60 h PBM (Fig. 1C-D).

*In situ hybridisation for detection and localisation of vitellogenin mRNAs* - To confirm PCR results, *in situ* hybridisation was used to provide a definitive demonstration of Vg transcripts accumulated in the ovaries.

The *in situ* hybridisation results confirmed that ovaries accumulate both CqVg1 (CPIJ010190/CPIJ010191; Fig. 2A) and CqVg2 (CPIJ001357/CPIJ001358; Fig. 2B) transcripts. The diffuse staining pattern throughout the follicle strongly suggests that the surveyed genes are transcribed and their transcripts accumulated by the cells of the follicular epithelium.

In insects, the follicular epithelium may perform multiple functions, including the synthesis of yolk proteins, production of ecdysone, and secretion of eggshell proteins as well as ligands responsible for determining antero-posterior and dorso-ventral axis of the developing embryo.^8^ ^,^ ^16^ ^,^ ^36^ ^-^ ^40^ Mosquito follicle cells have been associated with eggshell formation, but their involvement with other functions has not been reported to date.

Our results are the first to show that Vg genes are transcribed in mosquito ovaries, providing new insights on temporal and tissue expression regulation of these genes in *C. quinquefasciatus.* Additional studies are necessary to determine if the Vg transcripts are translated into proteins in the ovaries, and their potential contribution to the reproductive success of mosquitoes.
