# Supplementary material for: Vitellogenin genes are transcribed in Culex quinquefasciatus ovary
Source: Mem Inst Oswaldo Cruz. 2023 Jul 17;118:e220143. doi: 10.1590/0074-02760220143 (PMC10368008; doi:10.1590/0074-02760220143)
Supplement: Supplementary file 1 [file 1678-8060-mioc-118-e220143-s.pdf]

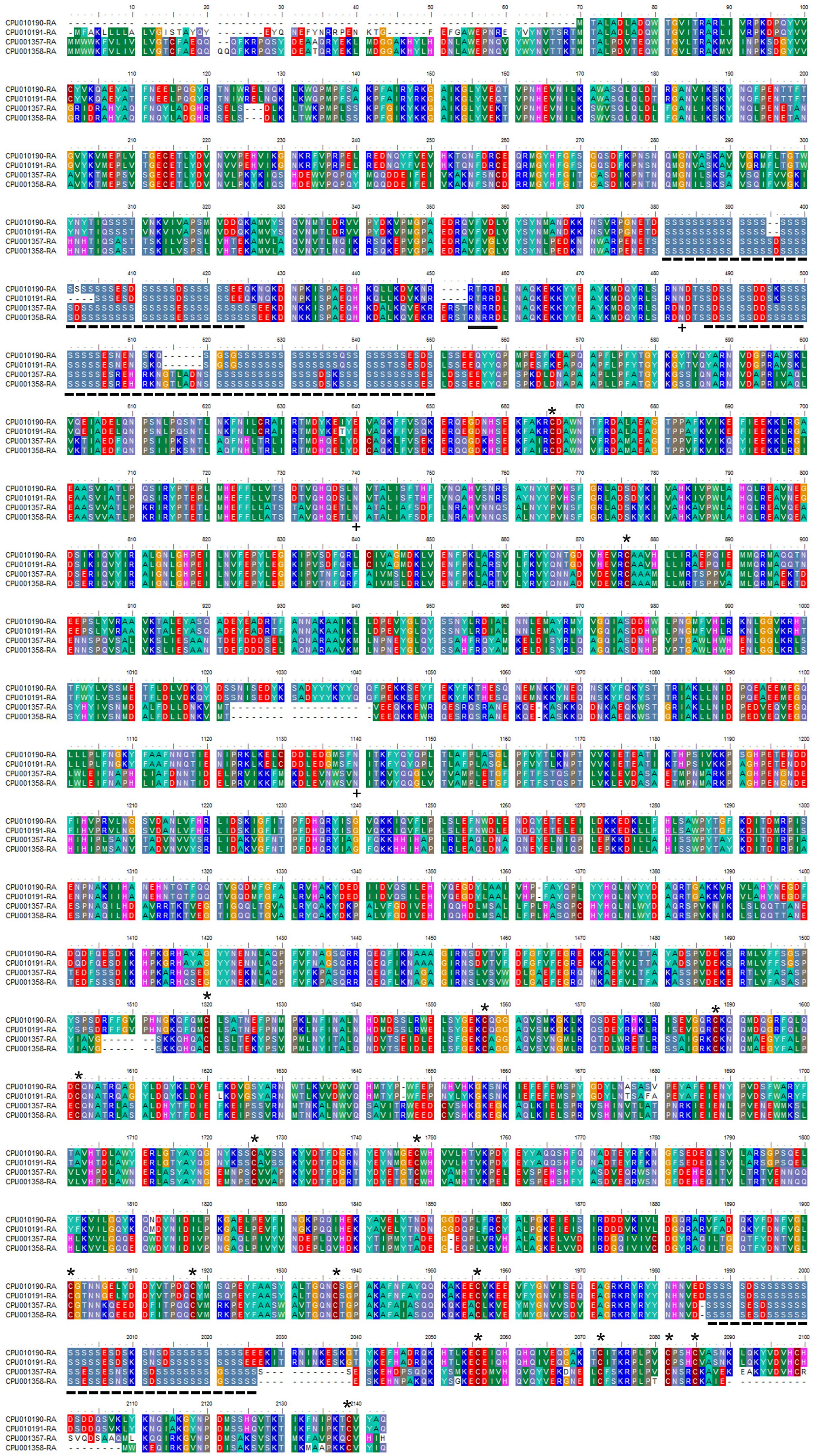

Fig. 1: a multiple alignment of the amino acid sequences of *Culex quinquefasciatus* vitellogenins. The underline indicates the cleavage region (RXRR) between the large and small subunits; asterisks point to the conserved cysteine residues; the dotted line shows the serine rich regions and symbol + indicate putative N-glycosylation sites in all sequences. On the left is shown the vitellogenins access number in VectorBase database. CPIJ001357; CPIJ001358; CPIJ010190 and CPIJ010191: vitellogenin-A1 precursor.

cc BY

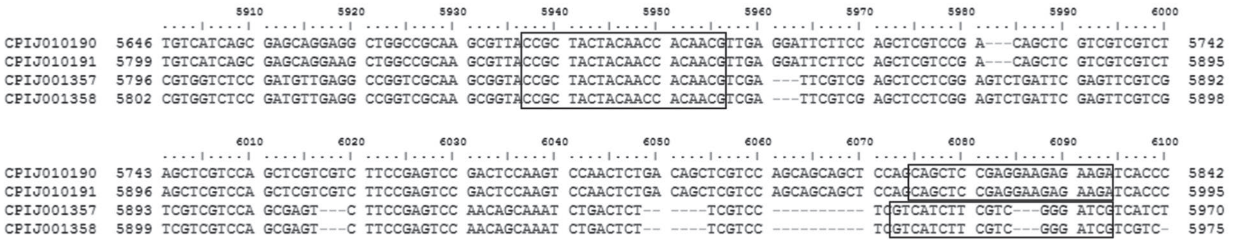

Fig. 2: alignment of the target sequences used to design the primers for amplification of the *Culex quinquefasciatus* vitellogenins transcripts. *C. quinquefasciatus* vitellogenin 1 gene (CqVg1) (CPIJ010190 and CPIJ010191) and *C. quinquefasciatus* vitellogenin 2 gene (CqVg2) (CPIJ001357 and CPIJ001358). Boxes indicate the sequences used to design forward and reverse primers.

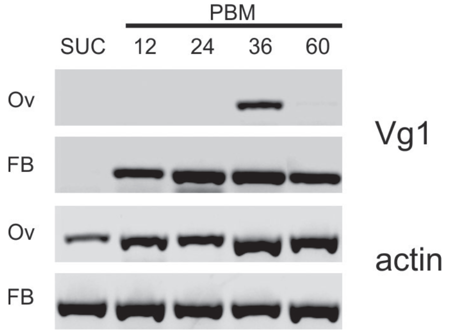

Fig. 3: reverse transcriptase polymerase chain reaction (RT-PCR) analyses of *Culex quinquefasciatus* vitellogenin 1 gene transcripts during the first gonotrophic cycle (SUC, 12 h, 24 h, 36 h, and 60 h post blood meal (PBM) in vitellogenic ovaries (Ov) and fat bodies (FB). SUC: non-blood fed females (sucrose). The top line is related to the ovary and the bottom line is related to the fat body for each expressed gene.
